# Radiological properties of neurological injury following acute type A aortic dissection repair

**DOI:** 10.1016/j.xjon.2023.06.005

**Published:** 2023-06-16

**Authors:** Jacob Ede, Karl Teurneau-Hermansson, Birgitta Ramgren, Marion Moseby-Knappe, Mårten Larsson, Johan Sjögren, Per Wierup, Shahab Nozohoor, Igor Zindovic

**Affiliations:** a Department of Clinical Sciences Lund, Lund University, Lund, Sweden; b Department of Cardiothoracic Surgery, Skåne University Hospital, Lund, Sweden; c Department of Radiology, Skåne University Hospital, Lund, Sweden; d Department of Neurology, Skåne University Hospital, Lund, Sweden

**Keywords:** aorta, dissection, embolism, stroke, watershed lesions

## Abstract

**Objective:**

The study objective was to assess the radiological properties of acute type A aortic dissection–related neurological injuries and identify predictors of neurological injury.

**Methods:**

Our single-center, retrospective, observational study included all patients who underwent acute type A aortic dissection repair between January 1998 and December 2021. Multivariable analyses and Cox regression were performed to identify predictors of embolic lesions, watershed lesions, neurological injury, 30-day mortality, and late mortality.

**Results:**

A total of 538 patients were included. Of these, 120 patients (22.3%) experienced postoperative neurological injury; 74 patients (13.8%) had postoperative stroke, and 36 patients (6.8%) had postoperative coma. The 30-day mortality was 22.7% in the neurological injury group versus 5.8% in the no neurological injury group (*P* < .001). We identified several independent predictors of neurological injury. Cerebral malperfusion (odds ratio, 2.77; 95% confidence interval, 1.53-5.00), systemic hypotensive shock (odds ratio, 1.97; 95% confidence interval, 1.13-3.43), and aortic arch replacement (odds ratio, 3.08; 95% confidence interval, 1.17-8.08) predicted embolic lesions. Diabetes mellitus (odds ratio, 5.35; 95% confidence interval, 1.85-15.42), previous cardiac surgery (odds ratio, 8.62; 95% confidence interval, 1.47-50.43), duration of hypothermic circulatory arrest (odds ratio, 1.05; 95% confidence interval, 1.01-1.08), cardiopulmonary bypass time (odds ratio, 1.01; 95% confidence interval, 1.00-1.01), ascending aortic/arch cannulation (odds ratio, 5.68; 95% confidence interval, 1.88-17.12), and left ventricular cannulation (odds ratio, 17.81; 95% confidence interval, 1.69-188.01) predicted watershed lesions. Retrograde cerebral perfusion (odds ratio, 0.28; 95% confidence interval, 0.01-0.84) had a protective effect against watershed lesions.

**Conclusions:**

In this study, we demonstrated that the radiological features of neurological injury may be as important as clinical characteristics in understanding the pathophysiology and causality behind neurological injury related to acute type A aortic dissection repair.

## Video Abstract

**Graphical abstract:**

**Figure:**
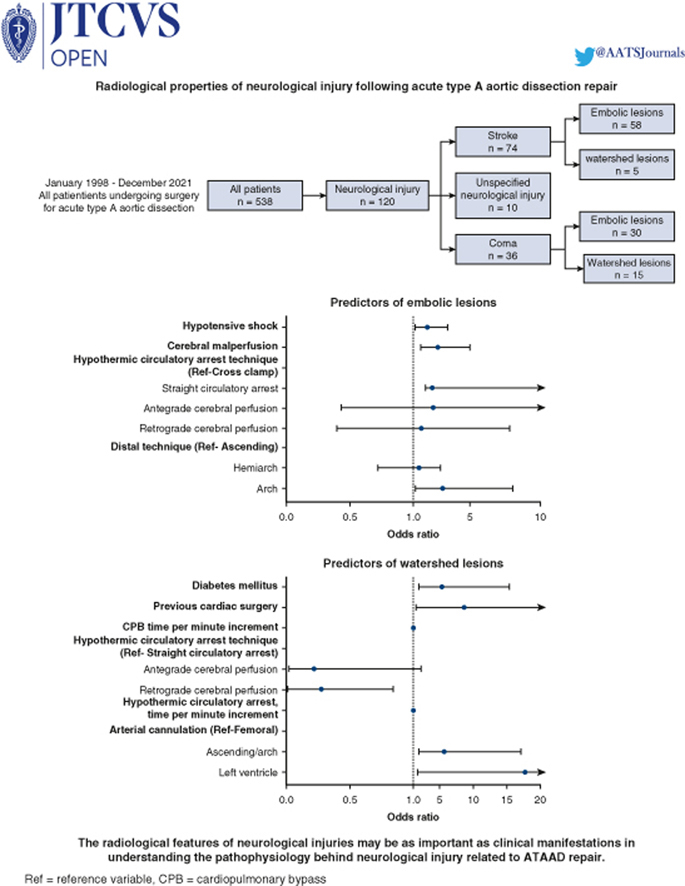

**Figure:**
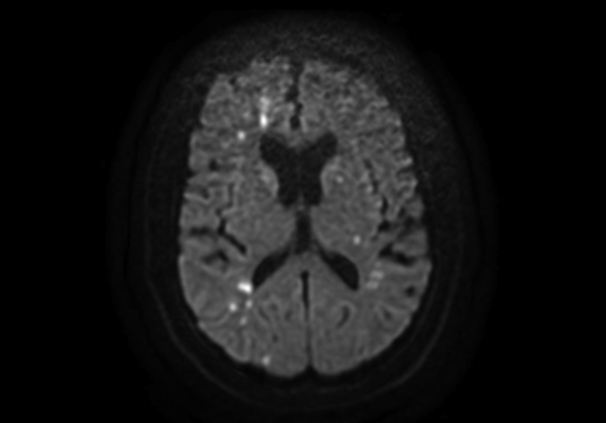
MRI of embolic lesions.

**Central Message**

The radiological features of neurological injuries may be as important as clinical manifestations in understanding the pathophysiology behind neurological injury related to ATAAD repair.

**Perspective**

Although neurological injuries after ATAAD repair are well studied, there are limited data on the radiological properties of these injuries. By assessment of available data, this study emphasized that radiological information may provide a better understanding of the mechanisms responsible for neurological injury after ATAAD repair.

Neurological injury associated with acute type A aortic dissection (ATAAD) and subsequent surgical repair is linked to a significant increase in mortality and morbidity and therefore poses a crucial clinical challenge.^1^, ^2^, ^3^, ^4^ Upon hospital admission, 6% to 20% of patients have neurological symptoms (ie, focal neurological deficits or altered state of consciousness).^1^^,^^2^^,^^4^, ^5^, ^6^, ^7^ The pathophysiological mechanisms of preoperative neurological injury include dissection of the supra-aortic blood vessels; embolization; or systemic hypotension due to shock, hypovolemia, severe aortic regurgitation, or cardiac tamponade.^8^

After ATAAD repair, 10% to 16% of patients experience postoperative stroke and 3% to 9% experience coma.^1^^,^^2^^,^^4^^,^^7^^,^^9^^,^^10^ Patients with postoperative neurological injury have short- and mid-term mortality rates twice those of patients with no signs of cerebral injury.^1^ Previously, stroke after ATAAD repair with deep hypothermic circulatory arrest was thought to be caused by hypoperfusion during the arrest, where time was the most important factor. However, recent data suggest that postoperative neurological injury could be the result of cerebral malperfusion, hypoperfusion, or embolism occurring at any time during the perioperative period.^8^ Surgical factors, including the arterial cannulation strategy, choice of cerebral perfusion during deep hypothermic circulatory arrest, extent of distal repair, and risk of residual intimal flaps in the downstream aorta or branch vessels are all associated with the risk of postoperative neurological injury.^1^^,^^3^^,^^6^^,^^9^

Previous studies on neurological injury after ATAAD repair have mainly relied on the clinical manifestation of neurological injury. Only one study detailed the radiological features of cerebral injuries associated with ATAAD repair, and it was limited by a small study population.^11^ The aim of this study was to assess the radiological properties of ATAAD-related neurological injuries and to identify predictors of these neurological injuries based on their radiological profile rather than their clinical manifestations.

## Materials and Methods

### Study Design

This study was a single-center, retrospective, observational study and included all patients who underwent ATAAD surgery between January 1998 and December 2021 at our institution. Data were prospectively entered into our departmental surgical database, and additional data were collected by retrospective chart review and radiological assessment. The study was approved by the Ethical Review Authority, Stockholm, Sweden (ref. 2021-01185, April 23, 2021). Individual patient consent was waived.

### Outcomes and Definitions

The primary outcome measures for this study were clinical neurological injury, embolic cerebral lesions, and watershed cerebral lesions. Clinical neurological injury was identified at wake up and was defined as focal neurological deficit or coma diagnosed by clinical assessment with or without radiological confirmation (computed tomography or magnetic resonance imaging [MRI]) with a symptom duration of more than 24 hours. Embolic lesions were defined as ischemia spread in the brain parenchyma, and watershed infarctions were defined as ischemic lesions occurring at the border zones between major cerebral artery territories resulting from cerebral hypoperfusion. Postoperative coma was defined as Glasgow Coma Scale motor score less than 6, 48 hours after termination of pharmacological sedation. Patients who were comatose postoperatively and regained consciousness with persisting stroke symptoms were regarded as coma patients. The radiological assessment was performed by a senior consultant neuroradiologist. Postoperative stroke was defined as focal neurological symptoms with a duration of more than 24 hours confirmed by a neurologist or with radiological findings correlating with the symptoms. If a neurological injury was presumed, a neurologist would be consulted. When classification was unclear, a neurologist reviewed the documentation of neurological symptoms. Hypotensive shock was defined as systolic blood pressure less than 90 mm Hg, clinical signs of hypotension, or the preoperative use of vasopressors or inotropes. Malperfusion was defined as clinical signs of end-organ ischemia, and cerebral malperfusion was defined as focal neurological deficit or altered state of consciousness before surgery.

### Surgical Technique

The surgical technique used at our institution has been described in detail.^12^ In summary, repair was performed using median sternotomy, cardiopulmonary bypass, and intermittent cardioplegic arrest. Cannulation site varied at the discretion of each surgeon. In a minority of cases, aortic crossclamp was used, but in general, the distal anastomosis, resection, and inspection of the aortic arch were performed under deep or moderate hypothermic circulatory arrest, with or without the use of antegrade cerebral perfusion (ACP) or retrograde cerebral perfusion (RCP). The decision to use RCP was based on surgeon preference, and there were no patient or clinical triggers. The use of ACP was limited to arch procedures or when the anticipated circulatory arrest exceeded 30 minutes. If a limited distal repair (ascending aortic arch or hemiarch repair) was feasible, this approach was favored at our institution, but the technique used depended on the location of the intimal tear and the extent of dissection. Aortic arch procedures entailed reimplantation of any supra-aortic branch. Aortic valve replacement or total root replacement was performed when the dissection involved the coronary ostia or aortic valve, or in the presence of an aortic root aneurysm. When required, the competence of the aortic valve was restored via subcommissural plication, commissural resuspension, or valvuloplasty. Concomitant procedures (eg, coronary artery bypass) were performed when required.

### Statistical Analysis

Categorical variables were presented as numbers and percentages. Continuous data were reported as median with interquartile range or mean ± standard deviation depending on the distribution of data. Chi-square test, Fisher exact test, 2-sample *t* tests, and Mann–Whitney *U* test were used for intergroup comparisons when appropriate. Patients who died intraoperatively were excluded from group analysis. Logistic regression was used for univariable and multivariable analyses to identify predictors of neurological injury and 30-day mortality. A *P* value of less than .1 in the univariable model was required for a variable to be included in the full model. Multicollinearity between continuous variables was assessed by linear regression using variance inflation factor. Spearman correlation was used for testing multicollinearity between categorical values and Pearson correlation between categorical and continuous values. Two variables with a variance inflation factor greater than 3.0 or correlation coefficient of greater than 0.5 were defined as being multicollinear. Multicollinearity was identified between any malperfusion and cerebral malperfusion and between clinical neurological injury and radiological neurological injury. Any malperfusion was our preferred variable when assessing 30-day mortality, whereas cerebral malperfusion was used for the analysis of neurological and radiological outcomes. In models where neurological injury was tested, neurological injury was used in the baseline model, but radiological and clinical properties were analyzed in separate models to generate the specific odds ratios (ORs) for these variables.

To reduce the number of variables in the multivariable analysis, we considered reoperation for bleeding as a marker of postoperative bleeding complications, postoperative renal replacement therapy as an indicator of renal injury, and hypotensive shock as an indicator of circulatory instability. Because the model relied on complete case analysis, lactate, postoperative myocardial infarction, fibrinogen, and recombinant factor VIIa were excluded due to numerous missing values. The analyses were performed using the Backward Wald method, and the variables remaining at the last step were reanalyzed using the Enter method to generate the final ORs. Goodness-of-fit of the multivariable model was evaluated using the Omnibus Tests of Model Coefficients and the Hosmer–Lemeshow test. Predictors of late mortality were assessed with Cox proportional hazard regression using stepwise backward elimination, including only 30-day survivors. The results of the logistic regression analyses are expressed as ORs and those of the Cox regression analysis as hazard ratios (HRs), both with 95% confidence intervals (CIs). ORs and HRs were illustrated using forest plots. Late survivals ±1 standard error were illustrated using the Kaplan–Meier method. All statistical analyses were conducted using IBM SPSS Statistics version 28.0 (IBM Corp).

## Results

### Study Population

**Figure E1:**
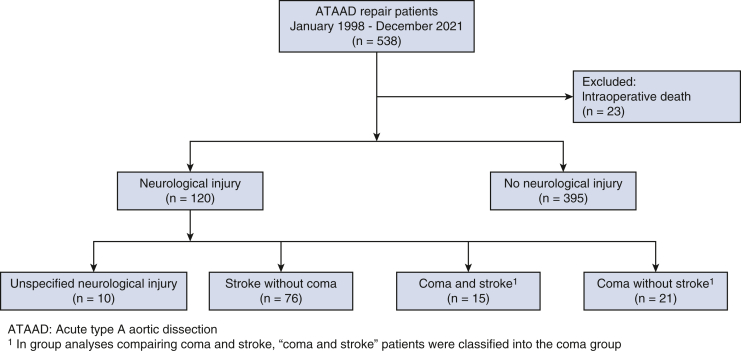
STROBE flow chart of patients in the study. *ATAAD*, Acute type A aortic dissection.

Between January 1998 and December 2021, 538 patients underwent ATAAD surgery at our institution, all of whom were included in the present study. Follow-up was conducted in January 2022 with a total of 3346 patient-years (median 5.1 [1.2-10.1], mean 6.2 ± 5.7). A total of 23 patients (4.3%) died intraoperatively. A total of 120 patients (22.3%) experienced postoperative neurological injury, of whom 74 patients (13.8%) had stroke and 36 patients (6.8%) had coma. A total of 15 patients (2.8%) were initially comatose and showed focal neurological symptoms after regaining consciousness. Ten patients (1.9%) had an unspecified neurological injury (Figure E1). A total of 78 patients (14.5%) presented with cerebral malperfusion.

A significantly higher proportion of patients with neurological injury had diabetes mellitus (23.3% vs 15.4%, *P* = .045) and higher preoperative creatinine (103 μmol/L vs 87 μmol/L, *P* < .001) and lactate (2.6 mmol/L vs 1.8 mmol/L, *P* < .001) (Table 1). These patients also presented significantly more often with syncope (26.7% vs 13.6%, *P* = .002), hypotensive shock (31.0% vs 20.5%, *P* = .030), and cardiac tamponade (23.0% vs 13.0%, *P* = .016) and more often had any malperfusion (50.8% vs 30.9%, *P* < .001) or cerebral malperfusion (30.0% vs 9.9%, *P* < .001). However, there was no difference between the groups in the prevalence of carotid artery dissection (57.4% vs 47.4%, *P* = .186).

**Table 1:** Baseline characteristics

Characteristic	All (538)	Neurological injury (120)	No neurological injury∗ (395)	*P*	Missing
Age, y	64.5 ± 11.6	65.4 ± 10.9	64.3 ± 11.9	.380	0
Female	192 (35.7)	43 (35.8)	144 (36.5)	.901	0
Hypertension	275 (51.5)	65 (54.2)	198 (50.1)	.438	0
Diabetes mellitus	96 (17.8)	28 (23.3)	61 (15.4)	.045	0
COPD	28 (5.2)	4 (3.3)	22 (5.6)	.327	0
History of smoking	171 (33.3)	41 (37.3)	122 (32.0)	.303	25 (4.6)
Coronary artery disease	30 (7.0)	6 (5.9)	22 (7.1)	.678	111 (20.6)
Known thoracic aneurysm	50 (11.7)	10 (10.0)	38 (12.3)	.528	112 (20.8)
Marfan syndrome	28 (5.2)	4 (3.3)	23 (5.8)	.284	0
Other connective tissue disease	5 (1.2)	1 (1.0)	4 (1.3)	1.000	111 (20.6)
Family history of dissection	22 (4.1)	4 (3.3)	18 (4.6)	.562	0
Previous cardiac surgery	13 (2.4)	3 (2.5)	10 (2.5)	1.000	2 (0.4)
Previous aortic surgery	16 (3.0)	3 (2.5)	12 (3.0)	1.000	1 (0.2)
Preoperative creatinine (μmol/L)	89 (73-111)	103 (77-127)	87 (72-105)	<.001	20 (3.7)
Preoperative lactate (mmol/L)	1.8 (1.2-3.3)	2.6 (1.3-4.7)	1.6 (1.2-2.8)	<.001	126 (23.3)
Syncope	76 (17.8)	27 (26.7)	42 (13.6)	.002	111 (20.6)
Hypotensive shock	100 (23.8)	31 (31.0)	62 (20.5)	.030	117 (21.7)
Preoperative cardiac arrest	19 (4.4)	6 (5.9)	10 (3.2)	.241	111 (20.6)
Cardiac tamponade	70 (16.4)	23 (23.0)	40 (13.0)	.016	112 (20.8)
Any malperfusion	194 (36.1)	61 (50.8)	122 (30.9)	<.001	0
Cerebral malperfusion	78 (14.5)	36 (30.0)	39 (9.9)	<.001	1 (0.2)
Carotid dissection				.186	72 (13.4)
None	236 (50.6)	46 (42.6)	179 (52.6)		
Unilateral	99 (21.2)	26 (24.1)	70 (20.6)		
Bilateral	131 (28.1)	36 (33.3)	91 (26.8)		
Intramural hematoma	62 (14.6)	12 (11.9)	49 (16.0)	.319	112 (20.8)
DeBakey type 1	419 (78.2)	95(79.8)	305 (77.4)	.616	2 (0.4)

Values are presented as mean ± standard deviation, n (%), or median (interquartile range). *COPD*, Chronic obstructive pulmonary disease.

∗ Patients who died intraoperatively were excluded from the group “No neurological injury” (n = 23).

Patients with postoperative neurological injury underwent extensive repair more often than the no neurological injury group (10.0% vs 4.3%, *P* = .017) (Table 2). They more often underwent surgery with straight circulatory arrest (52.5% vs 41.9%) or with ACP (9.2% vs 5.7%) and less often with RCP (35.0% vs 46.0%) (*P* = .046).

**Table 2:** Surgical data

Characteristic	All (538)	Neurological injury (120)	No neurological injury∗ (395)	*P*	Missing
Cardiopulmonary bypass time (min)	190 (150-235)	197 (153-251)	187 (148-225)	.050	2 (0.4)
Aortic crossclamp time (min)	85 (60-128)	90 (57-140)	83 (60-122)	.553	2 (0.4)
Hypothermic circulatory arrest technique				.043	8 (1.5)
Crossclamp	31 (5.8)	4 (3.3)	25 (6.4)		
SCA	233 (44.0)	63 (52.5)	163 (41.9)		
ACP	37 (7.0)	11 (9.2)	22 (5.7)		
RCP	229 (43.2)	42 (35.0)	179 (46.0)		
Hypothermic circulatory arrest time (min)	22 (17-30)	22 (16-32)	22 (17-30)	.902	43 (8.0)
Hypothermic circulatory arrest temperature (°C)	18.0 (16.8-20.0)	18.0 (17.0-20.4)	18.0 (16.7-20.0)	.602	4 (0.8)
Arterial cannulation				.595	3 (0.6)
Femoral	395 (73.6)	88 (73.3)	295 (74.7)		
Axillary	15 (2.8)	2 (1.7)	13 (3.3)		
Direct aortic	116 (21.6)	26 (21.7)	82 (20.8)		
Left ventricle/Unknown	9 (1.7)	3 (2.5)	5 (1.3)		
Distal surgical technique				.017	1 (0.2)
Ascending	438 (81.6)	89 (74.2)	334 (84.6)		
Hemiarch	66 (12.3)	19 (15.8)	44 (11.1)		
Arch	31 (5.8)	12 (10.0)	17 (4.3)		
Other/not completed	2 (0.4)	0 (0.0)	0 (0.0)		
Proximal surgical technique				.192	0 (0.0)
Supracoronary graft	385 (71.6)	90 (75.0)	283 (71.6)		
Bentall procedure	114 (21.2)	19 (15.8)	88 (22.3)		
Supracoronary + isolated AVR	27 (5.0)	8 (6.7)	18 (4.6)		
Root replacement with aortic valve repair	8 (1.5)	2 (1.7)	6 (1.5)		
Other/not completed	4 (0.7)	1 (0.8)	0 (0.0)		
Additional CABG	43 (8.0)	12 (10.0)	26 (6.6)	.210	0 (0.0)

Values are presented as mean ± standard deviation, n (%), or median (interquartile range). *SCA*, Straight circulatory arrest; *ACP*, antegrade cerebral perfusion; *RCP*, retrograde cerebral perfusion; *AVR*, aortic valve replacement; *CABG*, coronary artery bypass grafting.

∗ Patients who died intraoperatively were excluded from the group “No neurological injury” (n = 23).

### Radiological Properties of Neurological Injury

Both hemispheres were affected in more than half of patients (53.4%), but unilateral embolic injuries were more common in the right hemisphere than the left in patients with stroke and coma (70.4% vs 85.7%, *P* = .442). In patients with neurological injury, common carotid dissection was present on the left side in 6 patients (5.6%), right side in 20 patients (18.5%), and bilateral in 36 patients (33.3%). The middle cerebral artery was the most affected vascular territory in both groups (71.6% and 69.4%, respectively), but embolic lesions in the basilar artery territory were significantly more common in comatose patients (47.2% vs 20.3%, *P* = .009) as were watershed lesions (41.7% vs 6.8%, *P* < .001) and mixed lesions (30.6% vs 4.1%, *P* < .001) (Table 3).

**Table 3:** Radiological properties of neurological injury stratified by clinical presentation

Characteristic	All (n = 120)	Stroke (n = 74)	Coma∗ (n = 36)	*P*	Missing
Carotid artery dissection				.205	10 (8.3)
Left	6 (5.6)	5 (5.7)	0 (0)		
Right	20 (18.5)	12 (16.1)	6 (19.4)		
Both	36 (33.3)	28 (35.6)	8 (25.8)		
Postoperative imaging available				.091	20 (16.7)
CT	89 (89.0)	55 (74.3)	34 (94.4)		
MRI	11 (11.0)	10 (13.5)	1 (2.8)		
Nonacute ischemic lesions/white matter disease	24 (24.0)	16 (21.6)	8 (22.2)	.84	20 (16.7)
Acute ischemic lesions	92 (92.0)	59 (79.7)	33 (91.7)	.709	20 (16.7)
Embolic	88 (88.0)	58 (78.4)	30 (83.3)	.748	
Watershed	20 (20.0)	5 (6.8)	15 (41.7)	<.001	
Mixed	14 (14.0)	3 (4.1)	11 (30.6)	<.001	
Embolic lesion hemisphere				.699	20 (16.7)
Left	10 (10.0)	8 (10.8)	2 (5.6)		
Right	31 (31.0)	19 (25.7)	12 (33.3)		
Both	47 (47.0)	31 (41.9)	16 (44.4)		
Embolic lesion territory					20 (16.7)
Anterior	17 (17.0)	9 (12.2)	8 (22.2)	.253	
Media	78 (78.0)	53 (71.6)	25 (69.4)	.244	
Posterior	42 (42.0)	26 (35.1)	16 (44.4)	.581	
Basilar	32 (32.0)	15 (20.3)	17 (47.2)	.009	
Watershed lesion hemisphere				<.001	20 (16.7)
Left	3 (3.0)	1 (1.4)	2 (5.6)		
Right	5 (5.0)	2 (2.7)	3 (8.3)		
Both	12 (12.0)	2 (2.7)	10 (27.8)		
Mixed lesions hemisphere				.003	20 (16.7)
Left	2 (2.0)	0 (0)	2 (5.6)		
Right	4 (4.0)	1 (1.4)	3 (8.3)		
Both	8 (8.0)	2 (2.7)	6 (16.7)		

Values are presented as n (%). *CT*, Computed tomography; *MRI*, magnetic resonance imaging.

∗ Patients with coma and symptoms of focal neurological injuries (stroke) were categorized into the coma group.

### Independent Predictors of Neurological Injury

**Figure 1:**
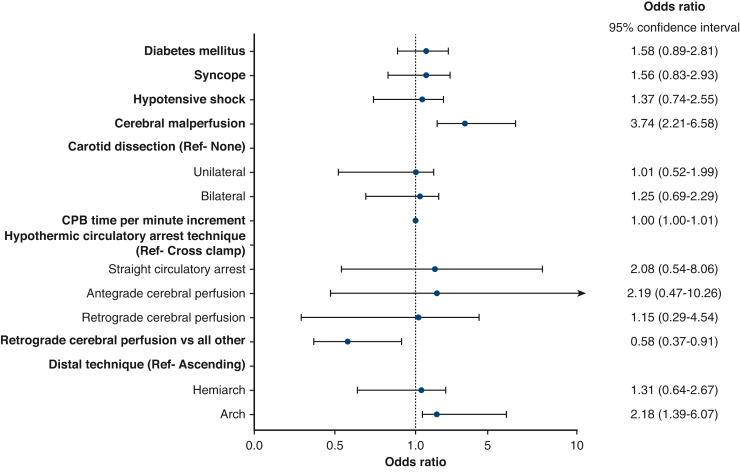
Forest plot illustrating the association with neurological injury. Values are expressed as OR with 95% CI. *CPB*, Cardiopulmonary bypass; *Ref*, reference variable.

Cerebral malperfusion (OR, 3.74; 95% CI, 2.12-6.58, *P* < .001) and cardiopulmonary bypass time (OR, 1.00; 95% CI, 1.00-1.01, *P* = .032) predicted neurological injuries, whereas RCP (OR, 0.58; 95% CI, 0.37-0.91, *P* = .015) had a protective effect (Figure 1 and Table E1).

### Independent Predictors of Embolic or Watershed Lesions

**Figure 2:**
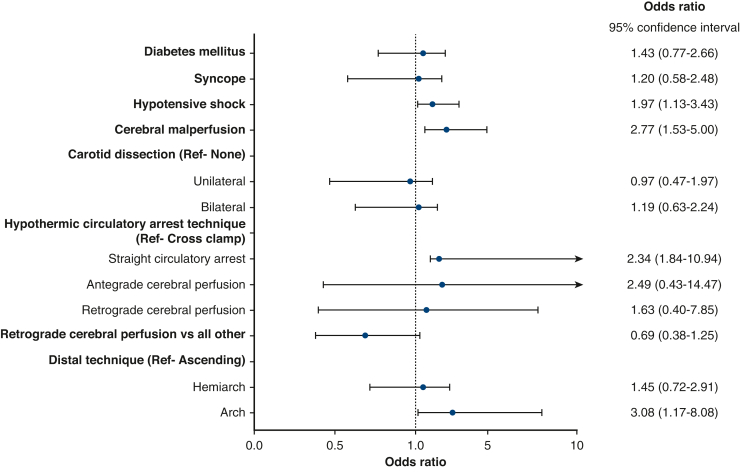
Forest plot illustrating the association with embolic lesions. Values are expressed as OR with 95% CI. *Ref*, Reference variable.

**Figure 3:**
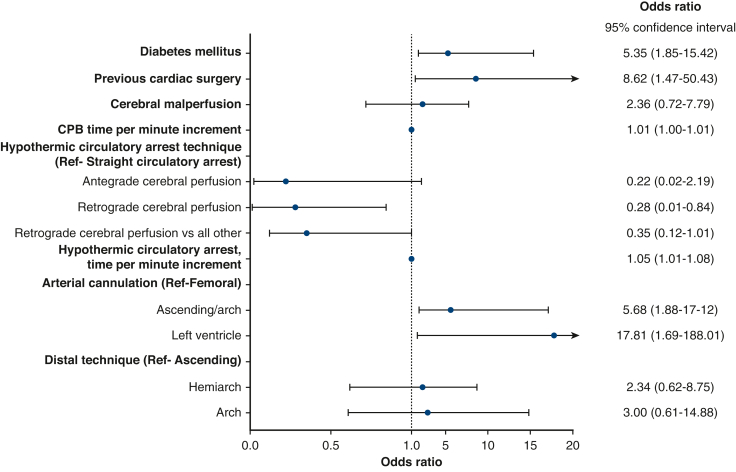
Forest plot illustrating the association with watershed lesions. Values are expressed as OR with 95% CI. *CPB*, Cardiopulmonary bypass; *Ref*, reference variable.

Cerebral malperfusion (OR, 2.77; 95% CI, 1.53-5.00, *P* < .001), systemic hypotensive shock (OR, 1.97; 95% CI, 1.13-3.43, *P* = .011), and aortic arch replacement (OR, 3.08; 95% CI, 1.17-8.08, *P* = .020) predicted embolic lesions (Figure 2 and Table E2). In turn, diabetes mellitus (OR, 5.35; 95% CI, 1.85-15.42, *P* = .002), previous cardiac surgery (OR, 8.62; 95% CI, 1.47-50.43, *P* = .017), hypothermic circulatory arrest time (OR, 1.05; 95% CI, 1.01-1.08, *P* = .005), cardiopulmonary bypass time (OR, 1.01; 95% CI, 1.00-1.01, *P* = .041), ascending aortic-/arch cannulation (OR, 5.68; 95% CI, 1.88-17.12, *P* = .002), and left ventricular cannulation (OR, 17.81; 95% CI, 1.69-188.01, *P* = .015) predicted watershed lesions, whereas RCP had a protective effect when compared with straight hypothermic circulatory arrest (OR, 0.28; 95% CI, 0.01-0.84, *P* = .023) (Figure 3 and Table E3).

### Postoperative Mortality

Overall, 30-day mortality was 13.7%. In patients who survived surgery, 30-day mortality was 9.7% (22.7% vs 5.8% in the neurological injury and no neurological injury group, respectively, *P* < .001).

Patients with neurological injury had significantly higher 24-hour bleeding volumes (730 mL vs 610 mL, *P* = .011) and received more blood products (packed red blood cells: 5 (2-12) units versus 3 (2-7) units, *P* < .001; plasma: 4 (0-8) units versus 3 (0-6) units, *P* = .035; platelets: 4 (4-6) units versus 4 (2-4) units, *P* < .001) (Table 4).

**Table 4:** Postoperative data

Characteristic	All (538)	Neurological injury (120)	No neurological injury∗ (395)	*P*	Missing
rFVIIa	102 (36.3)	30 (44.1)	72 (34.1)	.137	257 (47.8)
Fibrinogen (g)	4 (4-8)	6 (4-8)	4 (4-8)	.070	257 (47.8)
Bleeding first 24 h (mL)	640 (440-900)	730 (488-1345)	610 (420-855)	.011	262 (48.7)
Reoperated for bleeding	77 (14.3)	23 (19.2)	54 (13.7)	.142	1 (0.2)
PRBC units	4 (2-7.5)	5 (2-12)	3 (2-7)	<.001	133 (24.7)
Plasma units	3 (0-6)	4 (0-8)	3 (0-6)	.035	133 (24.7)
Platelet units	4 (2-6)	4 (4-6)	4 (2-4)	<.001	133 (24.7)
Ventilator >48 h	197 (39.7)	77 (66.4)	120 (31.6)	<.001	42 (7.8)
Renal replacement therapy	52 (10.2)	18 (15.0)	34 (8.7)	.046	27 (5.0)
Postoperative MI	20 (9.9)	8 (17.0)	12 (7.7)	.090	335 (62.3)
Multiple organ failure	8 (2.6)	5 (6.6)	3 (1.3)	.012	230 (42.8)
Postoperative peak CKMB (μg/L)	28.3 (18.1-55)	31.0 (22.8-65.0)	27.0 (17.0-53.4)	.015	207 (38.5)
Postoperative peak creatinine (μmol/L)	122 (91-201)	155 (104-257)	117 (88-182)	<.001	46 (8.6)
Postoperative peak lactate (mmol/L)	2.8 (2.0-3.8)	3.1 (2.2-5.0)	2.7 (2.0-3.5)	.028	263 (48.9)
Intraoperative death	23 (4.3)	NA	NA	NA	0
30-d mortality	73 (13.7)	27 (22.7)	23 (5.8)	<.001	7 (1.3)
In-hospital mortality	78 (14.6)	29 (24.4)	26 (6.6)	<.001	4 (0.7)

Values are presented as mean ± standard deviation, n (%), or median (interquartile range). *rFVIIa*, Recombinant factor VIIa; *PRBC*, packed red blood cells; *MI*, myocardial infarction; *CKMB*, creatine phosphokinase-MB; *NA*, not available.

∗ Patients who died intraoperatively were excluded from the group “No neurological injury” (n = 23).

### Independent Predictors of 30-Day Mortality

**Figure E2:**
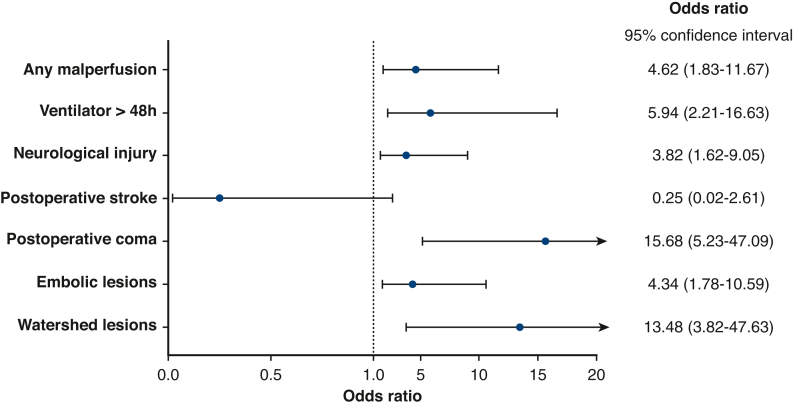
Forest plot illustrating the association with 30-day mortality. Values are expressed as OR with 95% CI.

We identified several independent predictors of 30-day mortality including malperfusion (OR, 4.62; 95% CI, 1.83-11.67, *P* = .011), neurological injury (OR, 3.82; 95% CI, 1.62-9.05, *P* < .001), coma (OR, 15.68; 95% CI, 5.23-47.09, *P* < .001), neurological injury with embolic lesions (OR, 4.34; 95% CI, 1.78-10.59, *P* < .001), and neurological injury with watershed lesions (OR, 13.48; 95% CI, 3.82-47.63, *P* < .001) (Figure E2 and Table E4).

### Late Survival

**Figure E3:**
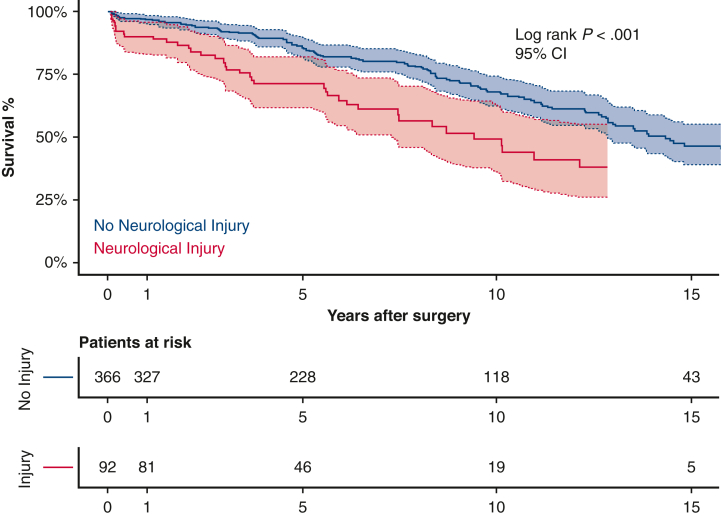
Kaplan–Meier survival curves of 30-day survivors demonstrating poorer survival in patients with neurological injury. *CI*, Confidence interval.

**Figure E4:**
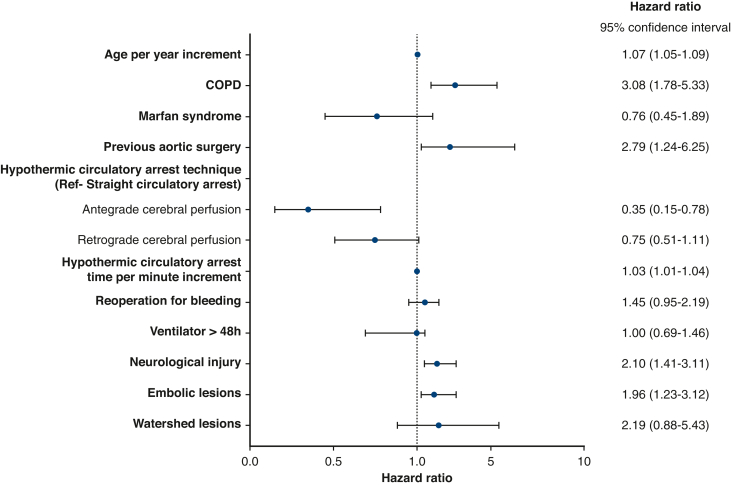
Forest plot illustrating independent predictors of late mortality in patients who survived to more than 30 days after surgery. Values are expressed as HR with 95% CI. *COPD*, Chronic obstructive pulmonary disease; *Ref*, reference variable.

Kaplan–Meier estimates of late survival in patients who survived 30 days postoperatively are demonstrated in Figure E3 and indicate that postoperative neurological injury is associated with significantly poorer survival at 1, 5, 10, and 15 years after ATAAD surgery (96.6% ± 0.9% vs 90.1% ± 3.1%, 86.3% ± 2.0% vs 71.1% ± 5.2%, 68.1% ± 3.1% vs 49.2% ± 6.7%, and 46.4% ± 4.1% vs 28.4 ± 7.9%, respectively; log rank *P* < .001). Independent predictors of late mortality are presented in Figure E4 and Table E5.

## Discussion

**Figure 4:**
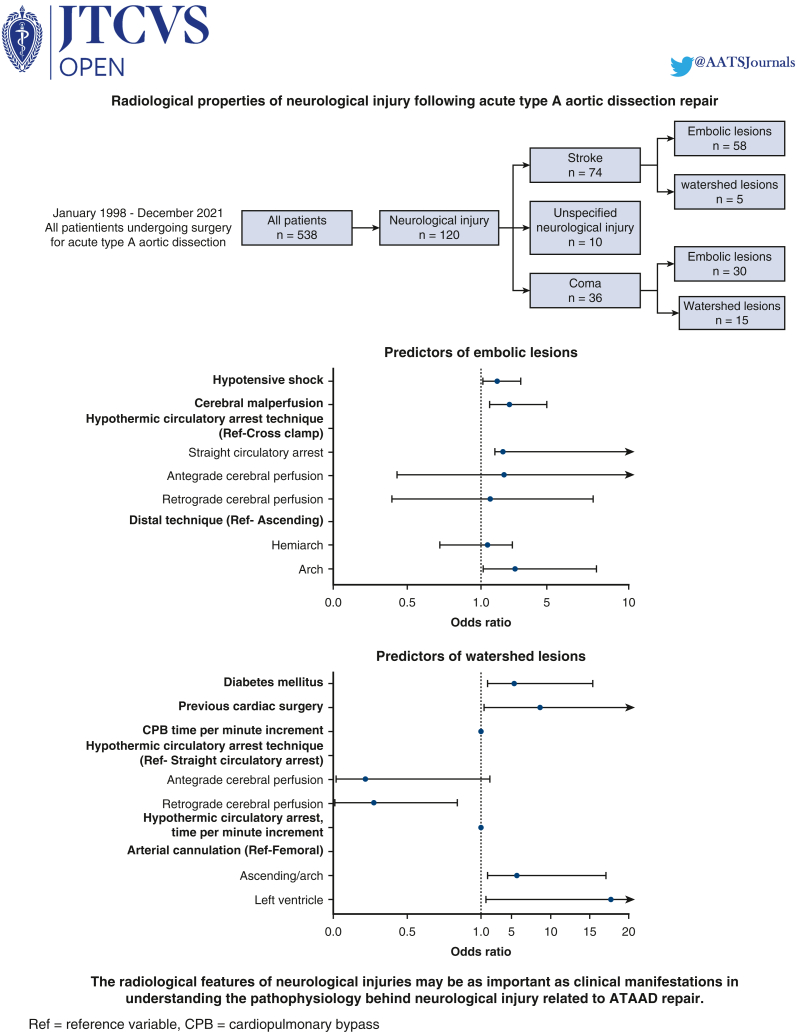
Significant predictors of embolic lesions and watershed lesions in patients undergoing surgical repair of ATAAD. Patients with coma and symptoms of focal neurological injuries (stroke) were categorized into the coma group. *CPB*, Cardiopulmonary bypass; *Ref*, reference variable; *ATAAD*, acute type A aortic dissection.

In this study, we showed that neurological injuries associated with ATAAD and subsequent surgical repair significantly increased postoperative mortality and morbidity. We also demonstrated that embolic injuries were more often observed in the right hemisphere, with the most common vascular territory being the middle cerebral artery. Embolic lesions in the basilar artery territory were more commonly associated with a postoperative comatose state, as were watershed lesions. As could be expected, preoperative cerebral malperfusion was associated with increased risk of postoperative neurological injury, but we could also show that RCP had a protective effect. Significant associations of embolic and watershed lesions are presented in Figure 4 and the Video Abstract.

Numerous factors have been reported to cause neurological injury after ATAAD surgery.^2^ Some factors are a consequence of the pathophysiological mechanisms of ATAAD, including arch vessel branch dissection and compromised cerebral blood flow, potential thromboembolism from clot formation in the exposed false lumen, systemic hypotension due to exsanguination, cardiac tamponade, or cardiogenic shock due to valve or coronary artery involvement. In addition, surgical factors including cannulation and cerebral protection strategies and extent of repair may all contribute to or even mitigate against neurologic injury.^1^, ^2^, ^3^^,^^9^ However, previous studies have relied on the clinical manifestation of neurological injury regardless of radiological appearance. We propose that assessing neurological injury based on its radiological properties rather than its clinical presentation may be more feasible for determining causality between preoperative and intraoperative factors and cerebral injury after ATAAD repair.

In our study, 22.3% of patients had postoperative neurological injury, 13.8% of whom had stroke and 6.8% were rendered comatose, regardless of preoperative state. The largest study included 9000 patients from the Society of Thoracic Surgeons Adult Cardiac Surgery Database reported a postoperative neurological complication rate of 13%. However, the proportion of patients with preoperative cerebral malperfusion was not presented, and patients who were in an unresponsive state at any time during the 24 hours preceding the procedure were excluded from the study.^9^ Recently, The Nordic Consortium for Acute Type A Aortic Dissection (NORCAAD) registry reported a stroke rate of 16%, and in the German Registry for Acute Aortic Dissection Type A (GERAADA) registry, 17% of patients had postoperative stroke.^1^^,^^6^ Most studies on ATAAD complications are self-reported by surgeons with the stroke definition including both clinical and radiological signs of neurological injury. This may lead to an underestimation of the true incidence of postoperative stroke because patients who die before radiological examinations are not included. Furthermore, a clinical examination performed by neurologists is more likely to detect minor neurological deficits.

Preoperative cerebral malperfusion was present in 14.5% of patients in this study, 8.5% of patients in the NORCAAD registry, and 20.5% of patients in the GERAADA database. In the present study, 30-day mortality was 13.7%, compared with 15.7% and 16.9% for NORCAAD and GERAADA, respectively.^1^^,^^6^ In line with previous findings, we demonstrated that cerebral malperfusion was an independent predictor of neurological injury. However, our study showed that less than half of patients with cerebral malperfusion had persistent neurological injuries after surgery.^1^^,^^4^ This indicates that, in a significant proportion of patients, cerebral malperfusion may be resolved by ATAAD repair.

Diabetes mellitus is associated with an increased risk of stroke in general with several proposed mechanisms, including vascular endothelial dysfunction and increased stiffness of the cerebral arteries.^13^ In our study, we showed that diabetes mellitus increases the risk of watershed injuries. It is reasonable to believe that patients with diabetes mellitus are more sensitive to perioperative systemic hypotension due to vasculopathy caused by the disease. Presumably, this could render diabetic patients more sensitive to shorter periods of circulatory arrest, both because of the general metabolic consequences of reduced blood flow and hypoperfusion leads to inadequate cooling of the cerebral parenchyma.

Previously, femoral cannulation has been demonstrated to increase the risk of perioperative stroke, and backwards flushing of embolic material from the dissected descending aorta has been suggested as an explanation.^9^^,^^14^ However, no randomized trials have been performed on cannulation strategies, and the results from the previous studies may be influenced by significant selection bias. The increased risk of stroke in patients with femoral cannulation may be related to surgical inexperience or hemodynamic instability.^14^ Axillary cannulation is well established and provides excellent results.^9^ However, the downside of this strategy is the time required to cannulate. In patients with unilateral postoperative neurological injury, we observed a higher frequency of embolic lesions in the right hemisphere compared with the left (73.6% vs 26.4%). The proposed mechanism of backward washing of embolic material using femoral cannulation most likely would lead to an increase of lesions in the left hemisphere. However, a post hoc analysis of patients with unilateral lesions, patients who had a femoral cannulation had 78.8% of lesions in the right hemisphere compared with 55.6% when other cannulation sites were used (*P* = .21).

Two previous studies contrast our findings. Fichadiya and colleagues^11^ did not identify any lateralization of unilateral strokes in patients with ATAAD, and a study of patients with cardioembolic stroke due to atrial fibrillation found that the lesions are equally distributed between the hemispheres, with a slightly higher proportion located in the left hemisphere.^15^^,^^16^ Embolic lesions in the right hemisphere might indicate that embolic material originates from the heart or ascending aorta. Because we only perform computed tomography or MRI on clinical suspicion of neurological injury, left-sided symptoms may be more easily recognized clinically than right-sided symptoms, which might increase the lateralization frequency in favor of the left hemisphere.^15^ In the absence of solid embolic material during ATAAD surgery and the higher occurrence of right-sided lesions, one may speculate that the lesions are caused by air trapped in the supra-aortic branch vessels during circulatory arrest. Although this is merely a speculation, this hypothesis may be supported by our finding that RCP has a protective effect on neurological injury. This is consistent with previous research showing that RCP during circulatory arrest reduces mortality and neurological injury when compared with straight circulatory arrest in both elective and emergency aortic surgery.^17^^,^^18^ Because RCP has little or no metabolic effect on the brain, it has been suggested that the protective effect is caused by topical cooling of the brain.^19^, ^20^, ^21^ However, Bonser and colleagues^19^ showed that when using ice packs as topical cooling (which is routinely used at our institution), the addition of RCP did not provide any change in nasopharyngeal temperature, suggesting that the cooling effect of RCP is limited. Therefore, the most likely protective effect of RCP is generated by the backward flow of blood, washing out air and other potential embolic material from the supra-aortic circulation.

The higher occurrence of right-sided lesions may be explained by our finding that the right common carotid artery was dissected more often than the left. This may contribute to the discrepancy between our study and the study by Fichadiya and colleagues,^11^ where no side difference was observed. Because the primary entry most often is located in the ascending aorta, it is logical that the dissection more often involves the first vessel taking off from the aortic arch.^6^

There is some evidence that in patients with cerebral malperfusion and occlusion of the carotid artery, cannulation with extra-anatomic bypass of the carotid artery might improve neurological outcomes.^22^^,^^23^ With the reservation that we did not distinguish between carotid artery dissection and occlusion, our findings that carotid artery dissection does not predict neurological injury and that most patients with cerebral malperfusion do not develop postoperative neurological injury suggest that carotid artery bypass should be considered only in highly selected cases.^1^

### Study Limitations

This study is limited by its retrospective design and by a database missing potentially significant variables. Because of the urgency of the diagnosis, preoperative brain imaging was not performed to confirm neurological injuries present before surgery. Not all cases of neurological injury could be radiologically confirmed, probably because of the low number of MRI examinations in this study. Furthermore, radiological embolic lesions could be the result of intimal flaps or local atherosclerotic plaques causing regional hypoperfusion instead of embolic. The primary assessment was not always performed using a standardized approach (ie, Glasgow Coma Scale or National Institutes of Health Stroke Scale). However, this study is strengthened by the assessment of all radiological examinations by a neuroradiologist and by the retrospective confirmation of neurological injury by a neurologist in cases where classification was unclear. Finally, because ACP was reserved for more complex procedures, our data regarding ACP are not scientifically reliable.

## Conclusions

This study showed that the radiological features may be as important as clinical manifestations in understanding the pathophysiology behind neurological injury related to ATAAD repair. Although embolic lesions more often occurred in the right hemisphere causing symptoms of stroke, basilary artery obstruction and watershed lesions were associated with postoperative coma. We also showed that cerebral malperfusion and diabetes mellitus play key parts in the development of neurological injury and that RCP has a neuroprotective effect.

### Conflict of Interest Statement

The authors reported no conflicts of interest.

The *Journal* policy requires editors and reviewers to disclose conflicts of interest and to decline handling or reviewing manuscripts for which they may have a conflict of interest. The editors and reviewers of this article have no conflicts of interest.

## Figures and Tables

**Table E1:** Univariable and multivariable logistic regression analysis for predictors of neurological injury

Characteristics	Univariable analysis (OR)	*P* value	Multivariable analysis (OR)	*P* value
Age per year increment	1.01 (0.99-1.03)	.380		
Female	0.97 (0.64-1.49)	.901		
Hypertension	1.18 (0.78-1.77)	.438		
Diabetes mellitus	1.67 (1.01-2.76)	.047	1.58 (0.89-2.81)	.117
COPD	0.59 (0.20-1.73)	.332		
History of smoking	1.26 (0.81-1.96)	.303		
Coronary artery disease	0.82 (0.32-2.09)	.678		
Known thoracic aortic aneurysm	0.79 (0.38-1.65)	.529		
Marfan syndrome	0.56 (0.19-1.65)	.290		
Other connective tissue disease	0.76 (0.08-6.88)	.807		
Heredity for aortic dissection	0.72 (0.24-2.18)	.563		
Previous cardiac surgery	0.99 (0.27-3.64)	.981		
Previous aortic surgery	0.82 (0.23-2.94)	.756		
Preoperative lactate per mmol increment	1.18 (1.07-1.29)	<.001		
Syncope	2.31 (1.34-4.00)	.003	1.56 (0.83-2.93)	.194
Hypotensive shock	1.75 (1.05-2.90)	.031	1.37 (0.74-2.55)	.191
All malperfusion	2.31 (1.53-3.51)	<.001		
Cerebral malperfusion	3.90 (2.34-6.51)	<.001	3.74 (2.12-6.58)	<.001
Carotid dissection				
None	Ref		Ref	
Unilateral	1.45 (0.83-2.52)	.193	1.01 (0.52-1.99)	.674
Bilateral	1.54 (0.93-2.55)	.093	1.25 (0.69-2.29)	.497
Intramural hematoma	0.71 (0.36-1.40)	.320		
DeBakey 1	1.16 (0.70-1.92)	.577		
Cardiopulmonary bypass time per minute increment	1.00 (1.00-1.01)	.032	1.00 (1.00-1.01)	.021
Aortic crossclamp time per minute increment	1.00 (1.00-1.01)	.366		
Hypothermic circulatory arrest technique				
Crossclamp	Ref		Ref	
SCA	2.42 (0.81-7.22)	.114	2.08 (0.54-8.06)	.138
ACP	3.13 (0.87-11.24)	.081	2.19 (0.47-10.26)	.542
RCP	1.47 (0.48-4.44)	.498	1.15 (0.29-4.54)	.096
RCP	0.59 (0.38-0.90)	.015	0.58 (0.37-0.91)	.017
Hypothermic circulatory arrest time per minute increment	1.01 (0.99-1.03)	.272		
Hypothermic circulatory arrest temperature per degree Celsius increment	0.98 (0.94-1.04)	.536		
Arterial cannulation				
Femoral	Ref			
Subclavian/brachiocephalic trunk	0.52 (0.11-2.33)	.389		
Ascending/arch	1.10 (0.66-1.81)	.696		
Left ventricle	2.01 (0.47-8.58)	.345		
Distal technique				
Ascending	Ref		Ref	
Hemiarch	1.62 (0.90-2.91)	.107	1.31 (0.64-2.67)	.641
Arch	2.65 (1.22-5.75)	.014	2.18 (0.79-6.07)	.930
Proximal technique				
Supracoronary graft	Ref			
Bentall procedure	0.68 (0.39-1.18)	.167		
Supracoronary + isolated AVR	1.40 (0.59-3.32)	.449		
Root replacement with aortic valve repair	1.05 (0.21-5.28)	.955		
Additional CABG	1.58 (0.77-3.23)	.213		
rFVIIa	1.52 (0.87-2.66)	.138		
Fibrinogen per g increment	1.08 (1.00-1.17)	.058		

Values are expressed as OR with 95% CI. *OR*, Odds ratio; *COPD*, chronic obstructive pulmonary disease; *Ref*, reference variable; *SCA*, straight circulatory arrest; *ACP*, antegrade cerebral perfusion; *RCP*, retrograde cerebral perfusion; *AVR*, aortic valve replacement; *CABG*, coronary artery bypass grafting; *rFVIIa*, recombinant factor VIIa.

**Table E2:** Univariable and multivariable logistic regression analysis for predictors of embolic neurological injury

Characteristics	Univariable analysis (OR)	*P* value	Multivariable analysis (OR)	*P* value
Age per year increment	1.00 (0.99-1.02)	.996		
Female	0.93 (0.57-1.49)	.750		
Hypertension	1.43 (0.90-2.27)	.128		
Diabetes mellitus	1.63 (0.94-2.83)	.085	1.43 (0.77-2.66)	.265
COPD	0.61 (0.18-2.08)	.431		
History of smoking	1.33 (0.81-2.16)	.257		
Coronary artery disease	0.84 (0.31-2.29)	.740		
Known thoracic aortic aneurysm	0.77 (0.35-1.72)	.529		
Marfan syndrome	0.58 (0.17-1.98)	.389		
Other connective tissue disease	0.98 (0.11-8.90)	.987		
Heredity for aortic dissection	0.31 (0.11-2.03)	.467		
Previous cardiac surgery	0.87 (0.19-3.97)	.852		
Previous aortic surgery	1.20 (0.33-4.35)	.781		
Preoperative lactate per mmol increment	1.09 (0.99-1.19)	.070		
Syncope	1.96 (1.10-3.51)	.023	1.20 (0.58-2.48)	.618
Hypotensive shock	2.00 (1.18-3.39)	.011	1.97 (1.13-3.43)	.016
All malperfusion	1.71 (1.08-2.71)	.023		
Cerebral malperfusion	2.93 (1.69-5.07)	<.001	2.77 (1.53-5.00)	<.001
Carotid dissection				
None	Ref		Ref	
Unilateral	1.09 (0.58-2.06)	.782	0.97 (0.47-1.97)	.922
Bilateral	1.50 (0.87-2.59)	.142	1.19 (0.63-2.24)	.593
Intramural hematoma	0.64 (0.30-1.36)	.243		
DeBakey 1	0.95 (0.55-1.65)	.862		
Cardiopulmonary bypass time per minute increment	1.00 (1.00-1.00)	.263		
Aortic crossclamp time per minute increment	1.00 (1.00-1.01)	.696		
Hypothermic circulatory arrest technique				
Crossclamp	Ref		Ref	
SCA	3.93 (0.91-17.11)	.680	2.34 (0.50-10.94)	.280
ACP	5.87 (1.17-29.57)	.032	2.49 (0.43-14.47)	.308
RCP	1.80 (0.40-8.02)	.440	1.63 (0.34-7.85)	.544
RCP	0.43 (0.26-0.71)	<.001	0.69 (0.38-1.25)	.223
Hypothermic circulatory arrest time per minute increment	0.99 (0.97-1.01)	.464		
Hypothermic circulatory arrest temperature per degree Celsius increment	1.02 (0.96-1.07)	.555		
Arterial cannulation				
Femoral	Ref			
Subclavian/brachiocephalic trunk	0.34 (0.04-2.65)	.343		
Ascending/arch	0.79 (0.62-1.88)	.787		
Left ventricle	0.57 (0.32-8.11)	.570		
Distal technique				
Ascending	Ref		Ref	
Hemiarch	1.69 (0.89-3.19)	.106	1.45 (0.72-2.91)	.273
Arch	2.06 (0.88-4.85)	.098	3.08 (1.17-8.08)	.020
Proximal technique				
Supracoronary graft	Ref			
Bentall procedure	0.61 (0.32-1.15)	.127		
Supracoronary + isolated AVR	1.32 (0.51-3.41)	.565		
Root replacement with aortic valve repair	1.60 (0.08-5.20)	.667		
Additional CABG	1.30 (0.58-2.95)	.524		
rFVIIa	1.10 (0.61-1.99)	.747		
Fibrinogen per g increment	1.06 (0.98-1.16)	.142		

Values are expressed as OR with 95% CI. *OR*, Odds ratio; *COPD*, chronic obstructive pulmonary disease; *Ref*, reference variable; *SCA*, straight circulatory arrest; *ACP*, antegrade cerebral perfusion; *RCP*, retrograde cerebral perfusion; *AVR*, aortic valve replacement; *CABG*, coronary artery bypass grafting; *rFVIIa*, recombinant factor VIIa.

**Table E3:** Univariable and multivariable logistic regression analysis for predictors of watershed neurological injury

Characteristics	Univariable analysis (OR)	*P* value	Multivariable analysis (OR)	*P* value
Age per year increment	1.01 (0.97-1.05)	.731		
Female	1.18 (0.47-2.93)	.727		
Hypertension	0.63 (0.25-1.56)	.316		
Diabetes mellitus	4.24 (1.70-10.58)	.002	5.35 (1.85-15.42)	.002
COPD	2.18 (0.48-9.94)	.314		
History of smoking	0.93 (0.35-2.48)	.879		
Coronary artery disease	0	.998		
Known thoracic aortic aneurysm	1.00 (0.22-4.51)	1		
Marfan syndrome	0.96 (0.12-7.37)	.949		
Other connective tissue disease	6.06 (0.64-57.38)	.116		
Heredity for aortic dissection	0	.998		
Previous cardiac surgery	4.88 (1.01-26.64)	.049	8.62 (1.47-50.43)	.017
Previous aortic surgery	1.81 (0.23-14.44)	.578		
Preoperative lactate per mmol increment	1.07 (0.92-1.25)	.371		
Syncope	1.06 (0.30-3.79)	.930		
Hypotensive shock	1.88 (0.67-5.21)	.229		
All malperfusion	2.23 (0.93-5.65)	.070		
Cerebral malperfusion	2.02 (0.71-5.73)	.187	2.36 (0.72-7.79)	.158
Carotid dissection				
None	Ref			
Unilateral	0.35 (0.08-1.57)	.169		
Bilateral	0.28 (0.17-1.66)	.530		
Intramural hematoma	0.65 (0.05-2.65)	.306		
DeBakey 1	1.63 (0.47-5.66)	.443		
Cardiopulmonary bypass time per minute increment	1.00 (1.00-1.01)	.044	1.01 (1.00-1.01)	.041
Aortic crossclamp time per minute increment	1.00 (0.99-1.01)	.649		
Hypothermic circulatory arrest technique				
SCA	Ref		Ref	
ACP	0.88 (0.05-14.65)	.926	0.22 (0.02-2.19)	.197
RCP	0.78 (0.09-6.73)	.822	0.28 (0.01-0.84)	.023
RCP	0.52 (0.20-1.41)	.203	0.35 (0.12-1.01)	.051
Hypothermic circulatory arrest time per minute increment	1.03 (1.00-1.11)	.060	1.05 (1.01-1.08)	.005
Hypothermic circulatory arrest temperature per degree Celsius increment	1.03 (0.933-1.14)	.571		
Arterial cannulation				
Femoral	Ref		Ref	
Subclavian/brachiocephalic trunk	0	.999	0	.999
Ascending/arch	3.36 (1.33-8.46)	.010	5.68 (1.88-17.12)	.002
Left ventricle	5.33 (0.60-47.49)	.134	17.81 (1.69-188.01)	.015
Distal technique				
Ascending	Ref		Ref	
Hemiarch	2.14 (0.68-6.78)	.197	2.34 (0.62-8.75)	.208
Arch	3.64 (0.98-13.58)	.054	3.00 (0.61-14.88)	.178
Proximal technique				
Supracoronary graft	Ref			
Bentall procedure	0.64 (0.18-2.25)	.490		
Supracoronary + isolated AVR	0.89 (0.11-7.01)	.914		
Root replacement with aortic valve repair	0	.999		
Additional CABG	2.32 (0.65-8.23)	.196		
rFVIIa	1.57 (0.24-12.62)	.579		
Fibrinogen per g increment	1.28 (1.02-1.60)	.033		

Values are expressed as OR with 95% CI. *OR*, Odds ratio; *COPD*, chronic obstructive pulmonary disease; *Ref*, reference variable; *SCA*, straight circulatory arrest; *ACP*, antegrade cerebral perfusion; *RCP*, retrograde cerebral perfusion; *AVR*, aortic valve replacement; *CABG*, coronary artery bypass grafting; *rFVIIa*, recombinant factor VIIa.

**Table E4:** Univariable and multivariable logistic regression analysis for predictors of 30-day mortality

Characteristics	Univariable analysis (OR)	*P* value	Multivariable analysis (OR)	*P* value
Age per year increment	1.02 (1.00-1.04)	.086		
Female	0.92 (0.55-1.54)	.741		
Hypertension	1.04 (0.63-1.70)	.878		
Diabetes mellitus	1.46 (0.80-2.64)	.215		
COPD	1.78 (0.69-4.54)	.231		
History of smoking	0.80 (0.46-1.39)	.424		
Coronary artery disease	1.19 (0.40-3.56)	.760		
Known thoracic aortic aneurysm	1.04 (0.42-2.59)	.932		
Marfan syndrome	0.47 (0.11-2.02)	.308		
Other connective tissue disease	0	.999		
Heredity for aortic dissection	0.99 (.29-3.43)	.988		
Previous cardiac surgery	4.19 (1.33-13.18)	.014		
Previous aortic surgery	1.46 (0.41-5.27)	.560		
Preoperative lactate	1.21 (1.11-1.34)	<.001		
Syncope	2.83 (1.48-5.42)	.002		
Hypotensive shock	1.85 (0.98-3.49)	.059		
Any malperfusion	1.90 (1.16-3.13)	.011	4.62 (1.83-11.67)	.015
Cerebral malperfusion	2.21 (1.21-4.02)	.009		
Carotid dissection				
None	Ref			
Unilateral	1.10 (0.55-2.22)	.791		
Bilateral	0.80 (0.40-1.61)	.535		
Intramural hematoma	0.35 (0.10-1.15)	.083		
DeBakey 1	1.01 (0.56-1.84)	.965		
Cardiopulmonary bypass time	1.01 (1.00-1.01)	<.001		
Aortic crossclamp time	1.00 (1.00-1.01)	.104		
Hypothermic circulatory arrest technique				
Crossclamp	Ref			
SCA	0.59 (0.22-1.55)	.283		
ACP	0.81 (0.23-2.81)	.736		
RCP	0.66 (0.25-1.73)	.393		
Hypothermic circulatory arrest time per minute increment	1.03 (1.01-1.04)	.005		
Hypothermic circulatory arrest temperature per degree Celsius increment	0.99 (0.94-1.05)	.826		
Arterial cannulation				
Femoral	Ref			
Subclavian/brachiocephalic trunk	1.02 (0.25-5.09)	.883		
Ascending/arch	1.94 (1.11-3.37)	.019		
Left ventricle	2.62 (0.51-13.35)	.248		
Distal technique				
Ascending	Ref			
Hemiarch	0.98 (0.44-2.17)	.964		
Arch	2.01 (0.85-4.87)	.125		
Proximal technique				
Supracoronary graft	Ref			
Bentall procedure	0.84 (0.44-1.60)	.589		
Supracoronary + isolated AVR	1.46 (0.53-4.03)	.464		
Root replacement with aortic valve repair	0.92 (0.11-7.62)	.937		
Other/not completed	19.29 (1.97-189.07)	.011		
Additional CABG	3.64 (1.82-7.32)	<.001		
rFVIIa	0.94 (0.36-2.45)	.905		
Fibrinogen per g increment	1.16 (1.03-1.31)	.014		
Reoperation for bleeding	2.01 (1.10-3.69)	.024		
Ventilator >48 h	5.29 (2.68-10.45)	<.001	5.94 (2.12-16.63)	<.001
Renal replacement therapy	2.35 (1.06-5.18)	.035		
Postoperative myocardial infarction	3.21 (0.94-11.01)	.064		
Postoperative lactate per mmol increment	1.33 (1.16-1.53)	<.001		
Neurological injury	4.67 (2.56-8.52)	<.001	3.82 (1.62-9.05)	<.001
Postoperative stroke	0.42 (0.15-1.12)	.103	0.25 (0.02-2.61)	.562
Postoperative coma	18.00 (8.41-38.55)	<.001	15.68 (5.23-47.09)	<.001
Nonacute lesions	1.40 (0.47-4.01)	.568		
Acute lesions	3.81 (2.00-7.72)	<.001		
Embolic lesions	3.40 (1.82-6.35)	<.001	4.34 (1.78-10.59)	.0095
Embolic territory				
Anterior	4.64 (1.55-13.84)	.006		
Media	3.09 (1.59-6.08)	.001		
Posterior	3.81 (1.72-8.40)	<.001		
Basilar	6.75 (3.00-15.18)	<.001		
Watershed lesions	8.92 (3.49-22.77)	<.001	13.48 (3.82-47.63)	<.001
Mixed lesions	16.27 (5.36-49.53)	<.001		

Values are expressed as OR with 95% CI. *OR*, Odds ratio; *COPD*, chronic obstructive pulmonary disease; *Ref*, reference variable; *SCA*, straight circulatory arrest; *ACP*, antegrade cerebral perfusion; *RCP*, retrograde cerebral perfusion; *AVR*, aortic valve replacement; *CABG*, coronary artery bypass grafting; *rFVIIa*, recombinant factor VIIa.

**Table E5:** Univariable and multivariable Cox regression analysis for predictors of late mortality

Characteristics	Univariable analysis (HR)	*P* value	Multivariable analysis (HR)	*P* value
Age per year increment	1.06 (1.05-1.08)	<.001	1.07 (1.05-1.09)	<.001
Female	1.09 (0.79-1.49)	.606		
Hypertension	1.20 (0.89-1.65)	.231		
Diabetes mellitus	0.97 (0.66-1.43)	.867		
COPD	3.06 (1.82-5.14)	<.001	3.08 (1.78-5.33)	<.001
History of smoking	1.13 (0.82-1.57)	.462		
Coronary artery disease	1.51 (0.78-2.91)	.222		
Known thoracic aortic aneurysm	1.50 (0.82-2.78)	.190		
Marfan syndrome	0.43 (0.19-0.97)	.042	0.76 (0.31-1.89)	.553
Other connective tissue disease	0.54 (0.08-3.91)	.545		
Heredity for aortic dissection	0.37 (0.12-1.15)	.084		
Previous cardiac surgery	1.35 (0.55-3.31)	.506		
Previous aortic surgery	3.66 (1.70-7.89)	<.001	2.79 (1.24-6.25)	.022
Preoperative lactate per mmol increment	1.05 (0.97-1.13)	.250		
Syncope	1.159 (0.67-2.01)	.598		
Hypotensive shock	1.37 (0.18-0.86)	2.17		
All malperfusion	1.20 (0.86-1.68)	.280		
Cerebral malperfusion	1.21 (0.75-1.95)	.442		
Carotid dissection				
None	Ref			
Unilateral	1.24 (0.80-1.91)	.334		
Bilateral	0.93 (0.60-1.45)	.753		
Intramural hematoma	0.83 (0.47-1.46)	.520		
DeBakey 1	0.92 (0.63-1.34)	.661		
Cardiopulmonary bypass time	1.00 (1.00-1.00)	.811		
Aortic crossclamp time	1.00 (1.00-1.00)	.775		
Hypothermic circulatory arrest technique				
Crossclamp	Ref		N/A	
SCA	2.47 (1.16-5.25)	.019	Ref	
ACP	1.16 (0.42-3.20)	.780	0.35 (0.15-0.78)	.004
RCP	2.01 (0.98-4.144)	.059	0.75 (0.51-1.11)	.074
Hypothermic circulatory arrest time per minute increment	1.02 (1.00-1.03)	.027	1.03 (1.01-1.04)	<.001
Hypothermic circulatory arrest temperature per degree Celsius increment	0.99 (−0.96-1.03)	.671		
Arterial cannulation				
Femoral	Ref			
Subclavian/brachiocephalic trunk	0.53 (0.17-1.66)	.273		
Ascending/arch	1.25 (0.86-1.82)	.245		
Distal technique				
Ascending	Ref			
Hemiarch	0.99 (0.61-1.61)	.972		
Arch	0.61 (0.31-1.21)	.156		
Proximal technique				
Supracoronary graft	Ref			
Bentall procedure	0.58 (0.38-0.87)	.006		
Supracoronary + isolated AVR	1.82 (0.98-3.37)	.059		
Root replacement with aortic valve repair	0.63 (0.16-2.58)	.526		
Additional CABG	1.34 (0.82-2.20)	.248		
rFVIIa	0.89 (0.48-1.63)	.697		
Fibrinogen per g increment	1.07 (0.99-1.16)	.082		
Reoperation for bleeding	2.06 (1.42-2.97)	<.001	1.45 (0.95-2.19)	.082
Ventilator >48 h	1.60 (1.17-2.21)	.004	1.00 (0.69-1.46)	.982
Renal replacement therapy	0.93 (0.54-1.61)	.794		
Postoperative myocardial infarction	2.24 (0.86-5.83)	.099		
Postoperative lactate per mmol increment	1.17 (1.01-1.35)	.041		
Neurological injury	1.98 (1.39-2.82)	<.001	2.10 (1.41-3.11)	<.001
Postoperative stroke	1.50 (0.81-2.77)	.198		
Postoperative coma	1.76 (0.86-3.60)	.119		
TIA	0.49 (0.97-3.48)	.473		
Nonacute lesions	2.05 (0.93-4.52)	.076		
Acute lesions	1.92 (1.28-2.91)	.002		
Embolic lesions	1.75 (1.14-2.68)	.010	1.96 (1.23-3.12)	.003
Watershed lesions	2.10 (0.92-4.75)	.076	2.19 (0.88-5.43)	.067
Mixed lesions	2.00 (0.64-6.29)	.237		

Values are expressed as HR with 95% CI. *HR*, Hazard ratio; *COPD*, chronic obstructive pulmonary disease; *Ref*, reference variable; *N/A*, not available; *SCA*, straight circulatory arrest; *ACP*, antegrade cerebral perfusion; *RCP*, retrograde cerebral perfusion; *AVR*, aortic valve replacement; *CABG*, coronary artery bypass grafting; *rFVIIa*, recombinant factor VIIa; *TIA*, transient ischemic attack.

## Supplementary Data

Video Abstract Dr Jacob Ede, Skåne University Hospital, presents the key findings of the present study. Video available at: https://www.jtcvs.org/article/S2666-2736(23)00160-2/fulltext.
